# Revisiting the healthy immigrant effect: Cardiovascular outcomes among US immigrants in a contemporary national cohort

**DOI:** 10.1016/j.ajpc.2026.101536

**Published:** 2026-03-24

**Authors:** Ashwin Kulshrestha, Nikhil Patel, Pranav Mellacheruvu, Rahul Aggarwal, Deepak L. Bhatt

**Affiliations:** a Icahn School of Medicine at Mount Sinai, New York, NY, USA; b Department of Medicine, Hospital of the University of Pennsylvania, Philadelphia, PA, USA; c Heart and Vascular Center, Brigham and Women’s Hospital, Harvard Medical School, Boston, Massachusetts, USA; d Mount Sinai Fuster Heart Hospital, Icahn School of Medicine at Mount Sinai, New York, NY, USA

**Keywords:** Immigrant health, Cardiovascular disparities, Health equity, Inflammation, NHANES

## 1 Introduction

Migration remains a defining feature of the United States, with immigrants today comprising over 15% of the US population [1]. Understanding immigrant health patterns is increasingly relevant to preventive care. Although cardiovascular disease (CVD) remains the leading cause of death nationally, disparities in risk and outcomes across demographic and socioeconomic groups underscore the importance of evaluating the social and structural determinants of cardiovascular health [2].

Epidemiologic studies have described a “healthy immigrant effect” by which first-generation immigrants exhibit more favorable health profiles and experience lower all-cause mortality despite lower average socioeconomic status compared with their non-immigrant counterparts [3,4]. While prior studies have largely focused on mediating risk factors, including hypertension, dyslipidemia, and diabetes, few have directly evaluated cardiovascular endpoints [[5], [6], [7]]. This represents a gap in our understanding. We therefore examined the cross-sectional association between immigrant status and self-reported CVD and assessed variation by duration of US residence.

## 2 Methods

We conducted a cross-sectional analysis of adults aged ≥20 years using the National Health and Nutrition Examination Survey (NHANES) August 2021 - August 2023 data. Immigrant status was defined by self-reported country of birth, and duration of US residence among immigrants was categorized as <10 years or ≥10 years. The primary outcome was self-reported cardiovascular disease, defined as a composite of myocardial infarction, stroke, coronary heart disease, angina, or heart failure. Survey weights were applied to account for the complex sampling design. Sequential survey-weighted logistic regression models were constructed: Model A adjusted for age, sex, and race and ethnicity; Model B additionally adjusted for socioeconomic variables (education, income-to-poverty ratio, and health insurance); and Model C further adjusted for behavioral and cardiometabolic variables (smoking, alcohol use, sedentary time, high-sensitivity C-reactive protein, total cholesterol, and blood pressure). Analyses were conducted using a complete-case approach; therefore, the analytic sample varied across models based on covariate availability. Age-standardized CVD prevalence by immigrant status was calculated using direct standardization to the 2000 US Census population.

## 3 Results

7793 participants were included in this analysis, of whom 25% were immigrants (Table 1). Immigrants were more likely to identify as Asian, Mexican American, or Other Hispanic (*p* < 0.001), while mean age and sex distribution were similar between groups. Meaningful differences in cardiovascular risk and sociodemographic profiles were observed. Immigrants had lower mean hs-CRP levels than non-immigrants (2.9 vs. 4.0 mg/L, *p* < 0.001), and higher total cholesterol levels (193 vs. 187 mg/dL, *p* = 0.034). Blood pressure measurements did not differ. Immigrants were more likely to have less than a ninth-grade education (14.0% vs. 1.3%; *p* < 0.001) and a household income below the federal poverty line (20.0% vs. 12.2%; *p* = 0.002). Significant differences were observed in health behaviors. Immigrants were less likely to report a history of smoking (26% vs. 41%, *p* < 0.001), as well as fewer minutes of daily sedentary time (298 vs. 383 min, *p* < 0.001). They reported higher average alcohol consumption, though this difference was not statistically significant (3.1 vs. 2.7 drinks/day, *p* = 0.074). Insurance coverage was substantially lower among immigrants, with 78% insured compared to 93% of non-immigrants (*p* < 0.001).

**Table 1:** Baseline Characteristics of Study Participants by Immigrant Status.

Variable	Non-Immigrant (Unweighted *N* = 6219)	Immigrant (Unweighted *N* = 1574)	p-value
Age, years	49.0	48.2	0.30
Sex			0.54
Male	2777 (48.7%)	702 (47.3%)	
Female	3442 (51.3%)	872 (52.7%)	
Race and Ethnicity			<0.001
Asian	67 (1.6%)	352 (24.7%)	
Black	855 (11.5%)	137 (9.1%)	
Mexican American	257 (4.8%)	288 (16.8%)	
Other Hispanic	256 (4.3%)	518 (31.2%)	
Other / Multiracial	442 (6.6%)	69 (3.9%)	
White	4342 (71.1%)	210 (14.5%)	
hs-CRP, mg/L	4.0	2.9	<0.001
HDL cholesterol, mg/dL	53.9	52.7	0.069
Total cholesterol, mg/dL	187.3	192.8	0.034
Systolic BP, mmHg	121.3	120.9	0.70
Diastolic BP, mmHg	75.3	74.6	0.31
Smoking status			<0.001
Yes (≥100 cigarettes)	2768 (40.7%)	464 (25.7%)	
No (<100 cigarettes)	3443 (59.1%)	1104 (74.2%)	
Refused	5 (0.02%)	2 (0.0%)	
Don’t know	3 (0.1%)	4 (0.1%)	
Education			<0.001
<9th grade	114 (1.3%)	259 (14.0%)	
9th–11th grade	486 (5.7%)	180 (9.3%)	
High School/GED	1458 (27.6%)	291 (22.0%)	
Some College	2035 (31.6%)	334 (20.5%)	
College Graduate or Above	2121 (33.9%)	504 (34.3%)	
Income			0.002
<100% FPL	757 (12.2%)	280 (20.0%)	
100–199% FPL	1052 (19.0%)	296 (22.3%)	
≥200% FPL	3458 (68.7%)	645 (57.7%)	
Health insurance			<0.001
Insured	5847 (93.4%)	1261 (78.4%)	
Uninsured	363 (6.5%)	305 (21.3%)	
Refused	3 (0.0%)	3 (0.07%)	
Don’t know	5 (0.15%)	4 (0.26%)	
Daily sedentary time, min	382.9	298.0	<0.001
Alcohol use, drinks/day	2.7	3.1	0.074
Coronary heart disease			0.014
Yes	336 (4.3%)	65 (2.8%)	
No	5856 (95.4%)	1502 (97.0%)	
Don’t know/Missing	27 (0.2%)	6 (0.2%)	
Angina			0.046
Yes	142 (1.8%)	39 (1.4%)	
No	6057 (98.1%)	1531 (98.0%)	
Don’t know	20 (0.2%)	4 (0.6%)	
Heart failure			0.006
Yes	292 (3.3%)	50 (1.7%)	
No	5914 (96.6%)	1520 (98.2%)	
Don’t know	13 (0.1%)	4 (0.1%)	
Myocardial infarction			0.023
Yes	283 (3.6%)	48 (2.1%)	
No	5927 (96.3%)	1521 (97.8%)	
Don’t know	9 (0.09%)	4 (0.08%)	
Stroke			0.27
Yes	308 (3.6%)	59 (3.3%)	
No	5897 (96.3%)	1506 (96.5%)	
Don’t know	14 (0.07%)	7 (0.17%)	
Adverse cardiovascular outcome (composite)			<0.001
Yes	824 (10.2%)	156 (7.0%)	
No	5395 (89.8%)	1416 (93.0%)	

FPL: Federal poverty line.

**Fig. 1:**
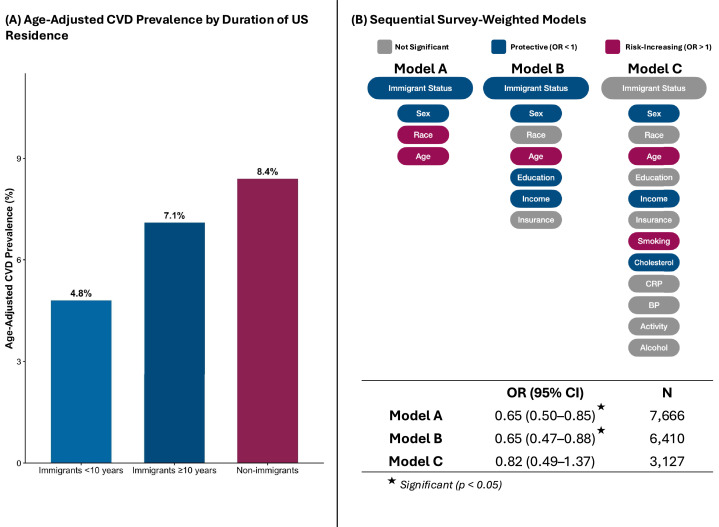
(A) Age-adjusted prevalence of self-reported CVD by immigrant status and duration of US residence. Prevalence was standardized to the 2000 US Census population. A graded pattern was observed, with lowest prevalence among immigrants residing <10 years and highest among non-immigrants. (B) Sequential survey-weighted logistic regression models evaluating the association between immigrant status and self-reported CVD. Models were adjusted for the listed variables. Odds ratios (95% CI) are shown. *p* < 0.05 indicates statistical significance.

The composite CVD outcome was positive in 10.2% of non-immigrants and 7.0% of immigrants overall (*p* < 0.001). In a three-group comparison, a graded pattern was observed (Fig. 1A). Crude prevalence was 2.7% among those residing in the US for <10 years, 8.8% in those residing ≥10 years, and 10.2% among non-immigrants (*p* = 0.002). After age-adjustment to the 2000 US population, a graded pattern by duration of residence persisted (4.8%, 7.1%, and 8.4%, respectively).

In sequential survey-weighted multivariable logistic regression models, immigrant status was independently associated with lower odds of cardiovascular events after adjustment for age, sex, and race and ethnicity (Model A; OR: 0.65, 95% CI: 0.50 – 0.85). This association remained similar after adjustment for education, income-to-poverty ratio, and health insurance (Model B; OR: 0.65, 95% CI: 0.47 – 0.88). In a fully adjusted model incorporating behavioral and cardiometabolic factors, the association was attenuated and no longer statistically significant (Model C; OR: 0.82, 95% CI: 0.49 – 1.37). Across models, increasing age was associated with higher odds of CVD, while female sex was associated with lower odds (Fig. 1B).

## 4 Discussion

In this cross-sectional analysis of a contemporary, nationally-representative dataset, immigrant status was associated with lower odds of self-reported CVD after demographic adjustment. This association persisted after adjustment for socioeconomic and healthcare access variables, and attenuated following inclusion of behavioral and cardiometabolic factors. Among immigrants, CVD prevalence revealed a graded pattern, with the lowest rates observed among immigrants residing in the US for less than 10 years and higher rates among those residing ≥10 years, approaching those of non-immigrants.

Our findings are consistent with prior literature describing a “healthy immigrant effect,” but should be interpreted cautiously. Because this study evaluated prevalent, self-reported CVD, multiple explanations for the observed association are possible. Lower prevalence of composite CVD among immigrants may reflect selective migration of healthier individuals, cohort effects related to country of migration, or return migration of individuals with advanced illnesses. Differences in measured risk factors, including smoking, sedentary behavior, and lipid profiles, may contribute to this effect, as suggested by attenuation in the fully adjusted model. However, the sequential modeling approach should be considered hypothesis-generating as it does not establish mediation. It remains possible that the association between immigrant status and CVD is explained by additional variables not included in the primary adjustment approach.

The graded pattern by duration of residence is consistent with prior literature suggesting that cardiovascular health profiles may evolve with increasing exposure to the US environment. However, this analysis did not include direct measures of acculturation, occupational exposures, dietary patterns, or neighborhood context. Therefore, while results are consistent with acculturation-related hypotheses, they do not establish causal effects of time spent in the US.

Insurance coverage was substantially lower among immigrants in our sample. Reduced access to healthcare among immigrants may increase likelihood of underdiagnosis, thus directly biasing self-reported prevalence. Simultaneously, improved healthcare access among non-immigrants may influence risk factor detection and control, potentially decreasing true disease burden over time. This analysis cannot distinguish between lower prevalence and lower detection rates.

This study has several strengths, including the use of contemporary nationally-representative data and standardized measures of cardiovascular risk. Several limitations warrant consideration. From a preventive cardiology perspective, the key unanswered question is whether immigrants experience lower risk factor burden, lower event incidence, or both. Because this study evaluates cross-sectional prevalent disease, it cannot assess incident risk or differentiate between differences in diagnosis or survival. Longitudinal cohorts with adjudicated cardiovascular outcomes are required to determine whether the observed patterns reflect true differences in event rates. Additionally, cardiovascular outcomes were self-reported and may be subject to misclassification, especially in the setting of differential healthcare access. Sequential models relied on complete-case analysis, resulting in reduced sample size in adjusted models, and potential selection bias if missingness was not random.

These findings provide contemporary evidence of differences in self-reported cardiovascular disease by immigrant status and duration of residence, consistent with prior descriptions of the healthy immigrant effect. As the US population becomes increasingly diverse, improved understanding of how cardiovascular risk evolves across the migration experience will be essential to preventive cardiology strategies and equitable healthcare delivery.

## Funding statement

No specific funding was applied for this project.

## Author agreement

This manuscript is original, has not been published previously, and is not under consideration elsewhere. All authors have approved the final manuscript and agree with its submission to AJPC.

## CRediT authorship contribution statement

**Ashwin Kulshrestha:** Writing – original draft, Formal analysis, Data curation, Conceptualization. **Nikhil Patel:** Writing – review & editing, Formal analysis, Data curation. **Pranav Mellacheruvu:** Writing – review & editing, Supervision. **Rahul Aggarwal:** Writing – review & editing. **Deepak L. Bhatt:** Writing – review & editing, Supervision.

## Declaration of competing interest

The authors declare the following financial interests/personal relationships which may be considered as potential competing interests:

Deepak L. Bhatt reports a relationship with Angiowave Imaging, Inc. that includes: board membership and equity or stocks. Deepak L. Bhatt reports a relationship with Antila Bioscience that includes: board membership. Deepak L. Bhatt reports a relationship with Bayer that includes: board membership and funding grants. Deepak L. Bhatt reports a relationship with Boehringer Ingelheim Corp USA that includes: board membership and funding grants. Deepak L. Bhatt reports a relationship with CellProthera SAS that includes: board membership and funding grants. Deepak L. Bhatt reports a relationship with Cereno Scientific that includes: board membership and funding grants. Deepak L. Bhatt reports a relationship with E-Star Biotech that includes: board membership. Deepak L. Bhatt reports a relationship with High Enroll that includes: board membership and equity or stocks. Deepak L. Bhatt reports a relationship with Janssen Pharmaceuticals Inc that includes: board membership and funding grants. Deepak L. Bhatt reports a relationship with Level Ex that includes: board membership. Deepak L. Bhatt reports a relationship with McKinsey that includes: board membership. Deepak L. Bhatt reports a relationship with Medscape Cardiology that includes: board membership. Deepak L. Bhatt reports a relationship with Merck & Co Inc that includes: board membership and funding grants. Deepak L. Bhatt reports a relationship with NirvaMed that includes: board membership. Deepak L. Bhatt reports a relationship with Novo Nordisk that includes: board membership and funding grants. Deepak L. Bhatt reports a relationship with Repair Biotechnologies Inc. that includes: board membership. Deepak L. Bhatt reports a relationship with Stasys that includes: board membership. Deepak L. Bhatt reports a relationship with SandBox AQ that includes: board membership and equity or stocks. Deepak L. Bhatt reports a relationship with Tourmaline Bio Inc that includes: board membership. Deepak L. Bhatt reports a relationship with Viatris that includes: board membership. Deepak L. Bhatt reports a relationship with Bristol-Myers Squibb Company that includes: board membership and equity or stocks. Deepak L. Bhatt reports a relationship with DRS.LINQ that includes: board membership and equity or stocks. Deepak L. Bhatt reports a relationship with Alnylam Pharmaceuticals Inc that includes: consulting or advisory and funding grants. Deepak L. Bhatt reports a relationship with Altimmune Inc that includes: consulting or advisory. Deepak L. Bhatt reports a relationship with Broadview Ventures Inc that includes: consulting or advisory. Deepak L. Bhatt reports a relationship with Corcept Therapeutics Inc that includes: consulting or advisory. Deepak L. Bhatt reports a relationship with Corsera that includes: consulting or advisory. Deepak L. Bhatt reports a relationship with GlaxoSmithKline that includes: consulting or advisory. Deepak L. Bhatt reports a relationship with Hims that includes: consulting or advisory. Deepak L. Bhatt reports a relationship with SERB that includes: consulting or advisory. Deepak L. Bhatt reports a relationship with SFJ that includes: consulting or advisory. Deepak L. Bhatt reports a relationship with Summa Therapeutics that includes: consulting or advisory. Deepak L. Bhatt reports a relationship with Worldwide Clinical Trials that includes: consulting or advisory. Deepak L. Bhatt reports a relationship with Acesion Pharma that includes: funding grants. Deepak L. Bhatt reports a relationship with Assistance Publique-Hôpitaux de Paris that includes: funding grants. Deepak L. Bhatt reports a relationship with Baim Institute for Clinical Research that includes: funding grants. Deepak L. Bhatt reports a relationship with Boston Scientific Corporation that includes: funding grants. Deepak L. Bhatt reports a relationship with Cleveland Clinic that includes: funding grants. Deepak L. Bhatt reports a relationship with Contego Medical Inc that includes: funding grants. Deepak L. Bhatt reports a relationship with Duke Clinical Research Institute that includes: funding grants. Deepak L. Bhatt reports a relationship with Mayo Clinic that includes: funding grants. Deepak L. Bhatt reports a relationship with Icahn School of Medicine at Mount Sinai that includes: funding grants. Deepak L. Bhatt reports a relationship with Novartis that includes: funding grants. Deepak L. Bhatt reports a relationship with Population Health Research Institute that includes: funding grants. Deepak L. Bhatt reports a relationship with Rutgers University that includes: funding grants. Deepak L. Bhatt reports a relationship with Arnold and Porter law firm that includes: paid expert testimony. Deepak L. Bhatt reports a relationship with Canadian Medical and Surgical Knowledge Translation Research Group that includes: funding grants. Deepak L. Bhatt reports a relationship with CSL Behring that includes: funding grants and speaking and lecture fees. Deepak L. Bhatt reports a relationship with Engage Health Media that includes: speaking and lecture fees. Deepak L. Bhatt reports a relationship with Medtelligence ReachMD that includes: speaking and lecture fees. Deepak L. Bhatt reports a relationship with MJH Life Sciences that includes: speaking and lecture fees. Deepak L. Bhatt reports a relationship with Oakstone CME that includes: speaking and lecture fees. Deepak L. Bhatt reports a relationship with Philips that includes: speaking and lecture fees. Deepak L. Bhatt reports a relationship with WebMd that includes: speaking and lecture fees. Deepak L. Bhatt reports a relationship with Wiley that includes: speaking and lecture fees. Deepak L. Bhatt reports a relationship with Added Health that includes: equity or stocks. Deepak L. Bhatt reports a relationship with Abbott that includes: funding grants. Deepak L. Bhatt reports a relationship with Afimmune that includes: funding grants. Deepak L. Bhatt reports a relationship with Amarin Pharma Inc that includes: funding grants. Deepak L. Bhatt reports a relationship with Amgen Inc that includes: funding grants. Deepak L. Bhatt reports a relationship with AstraZeneca that includes: funding grants. Deepak L. Bhatt reports a relationship with AtriCure Inc that includes: funding grants. Deepak L. Bhatt reports a relationship with Chiesi that includes: funding grants. Deepak L. Bhatt reports a relationship with Cleerly Inc that includes: funding grants. Deepak L. Bhatt reports a relationship with Faraday Pharmaceuticals that includes: funding grants. Deepak L. Bhatt reports a relationship with Fractyl Health, Inc. that includes: funding grants. Deepak L. Bhatt reports a relationship with Idorsia that includes: funding grants. Deepak L. Bhatt reports a relationship with Javelin that includes: funding grants. Deepak L. Bhatt reports a relationship with Lexicon Pharmaceuticals, Inc. that includes: funding grants. Deepak L. Bhatt reports a relationship with Lilly that includes: funding grants. Deepak L. Bhatt reports a relationship with Medtronic that includes: funding grants. Deepak L. Bhatt reports a relationship with MiRUS that includes: funding grants. Deepak L. Bhatt reports a relationship with Moderna Inc that includes: funding grants. Deepak L. Bhatt reports a relationship with Pfizer that includes: funding grants. Deepak L. Bhatt reports a relationship with PhaseBio Pharmaceuticals Inc that includes: funding grants. Deepak L. Bhatt reports a relationship with Regeneron Pharmaceuticals Inc that includes: funding grants. Deepak L. Bhatt reports a relationship with Reid Hoffman Foundation that includes: funding grants. Deepak L. Bhatt reports a relationship with Roche that includes: funding grants. Deepak L. Bhatt reports a relationship with Sanofi that includes: funding grants. Deepak L. Bhatt reports a relationship with Stasys that includes: funding grants. Deepak L. Bhatt reports a relationship with 89bio Inc that includes: funding grants. Deepak L. Bhatt has patent Sotagliflozin issued to named on a patent for sotagliflozin assigned to Brigham and Women’s Hospital who assigned to Lexicon; neither I nor Brigham and Women’s Hospital receive any income from this patent);. Clinical Cardiology (Deputy Editor, unpaid); Progress in Cardiovascular Diseases (Deputy Editor, unpaid). Rahul Aggarwal participates in research funded by the Bristol Myers Squibb-Pfizer alliance, Novartis, Amarin, Cleerly, and Lexicon. He is a consultant for Lexicon, Bayer, Viatris, and Amarin. He has served on an advisory board for Bayer. If there are other authors, they declare that they have no known competing financial interests or personal relationships that could have appeared to influence the work reported in this paper.

## References

[1] (2025). https://www.pewresearch.org/.

[2] Tejada-Vera B., Bastian B.A., Curtin S.C. (2025). Deaths: leading causes for 2023. Natl Vital Stat Rep.

[3] Minhas A.M.K., Talha K.M., Abramov D., Johnson H.M., Antoine S., Rodriguez F. (2024). Racial and ethnic disparities in cardiovascular disease: analysis across major US national databases. J Natl Med Assoc.

[4] Singh G.K., Siahpush M. (2002). Ethnic-immigrant differentials in health behaviors, morbidity, and cause-specific mortality in the United States: an analysis of two national databases. Hum Biol.

[5] Guadamuz J.S., Kapoor K., Lazo M., Eleazar A., Yahya T., Kanaya A.M (2021). Understanding immigration as a social determinant of health: cardiovascular disease in Hispanics/Latinos and South Asians in the United States. Curr Atheroscler Rep.

[6] Commodore-Mensah Y., Ukonu N., Obisesan O., Aboagye J.K., Agyemang C., Reilly C.M. (2016). Length of residence in the United States is associated with a higher prevalence of cardiometabolic risk factors in immigrants: a contemporary analysis of the National Health Interview Survey. J Am Heart Assoc.

[7] Commodore-Mensah Y., Selvin E., Aboagye J., Turkson-Ocran R.A, Li X., Himmelfarb C.D (2018). Hypertension, overweight/obesity, and diabetes among immigrants in the united states: an analysis of the 2010–2016 national health interview survey. BMC Public Health.

